# Alkali treated antioxidative crude polysaccharide from *Russula alatoreticula* potentiates murine macrophages by tunning TLR/NF-κB pathway

**DOI:** 10.1038/s41598-018-37998-2

**Published:** 2019-02-08

**Authors:** Somanjana Khatua, Krishnendu Acharya

**Affiliations:** Molecular and Applied Mycology and Plant Pathology Laboratory, Centre of Advanced Study, Department of Botany, University of Calcutta, 35, Ballygunge Circular Road, Kolkata, 700019 West Bengal India

## Abstract

In our previous research, *Russula alatoreticula* was demonstrated as a novel species, ethnic myco-food and reservoir of hot water extractable polysaccharides. However, residue after the hydrothermal process still offer plenty of medicinal carbohydrates that could easily be extracted by using alkali solvent. Thus, the present work was attempted to prepare crude polysaccharide using remainder of the conventional method and subsequently a β-glucan enriched fraction, RualaCap, was isolated. The bio-polymers displayed pronounced therapeutic efficacy as evident by radical scavenging, chelating ability, reducing power and total antioxidant capacity. In addition, strong immune-enhancing potential was also observed indicated by augmentation in macrophage viability, phagocytic uptake, nitric oxide (NO) production and reactive oxygen species (ROS) synthesis. Alongside, the polysaccharides effectively triggered transcriptional activation of Toll like receptor (TLR)-2, TLR-4, nuclear factor kappa B (NF-κB), cyclooxygenase (COX)-2, inducible nitric oxide synthase (iNOS), tumor necrosis factor (TNF)-α, Iκ-Bα, interferon (IFN)-γ and interleukin (IL)-10 genes explaining mode of action. Taken together, our results signify possibility of RualaCap as a potent nutraceutical agent and enhance importance of *R*. *alatoreticula* especially in the field of innate immune stimulation.

## Introduction

*Russula alatoreticula* has recently been established as a new species (Russulaceae, Basidiomycota) after thorough micro and macro-morphological studies followed by molecular systematics aspect. The taxon naturally grows in lateritic region of West Bengal, India particularly during rainy time forming ectomycorrhizal relationship with *Shorea robusta*. The macrofungus is customarily called “murgi chhatu” (“murgi” means hen and “chhatu” depicts mushroom) due to its striking red colour basidiocarps resembling comb of hen. It is noteworthy that tribal people are accustomed with the species being nutritional supplement that purportedly enhances overall immunity. Such ethno-mycological claim has scientifically been proved in our previous research demonstrating bioactive potential of hot water extracted polysaccharide from *R*. *alatoreticula*^1^.

In general, hydrothermal reflux is the most widely followed technique for polysaccharide isolation; although the method is associated with longer fractionation time and higher temperature (100 °C) demanding elevated energy requirement^2^. Besides, the process results in lower yield such that even after 5–6 h extraction the leaching efficacy ranges between 1–5%^3^. Thus, the observation clearly indicates that leftover residue still contain plenty of carbohydrate as it is the most abundant component of a mushroom^4^. In this scenario, alkali solution is considered as a popular approach as it breaks fungal cell wall from outer layer to inner due to strong pH condition^5^. Therefore, the solvent enhances recovery percentage particularly acidic and high molecular weight polysaccharides as they are less soluble in hot water^6^. Consequently, medium to large molecular weight biopolymers have been reported to exhibit diverse medicinal properties such as immune boosting and antioxidant activities^7^.

Immunotherapy by means of enhancement of host immune machinery is gaining additional significance in the present time as frequency of cancer, infectious diseases and immune-deficiencies are increasing. During the treatment, innate immunity is mainly targeted for stimulation being the front line of defence against pathogen attack^8^. In this context, macrophages are generally aimed for modification since they function as key orchestrator between two interactive arms of our defence mechanism, innate and adaptive. These mononuclear cells identify pathogens through their multiple cell surface receptors and among them TLRs play important role to activate the non-specific immunity. Upon stimulation TLRs drive downstream pathway mediated by a transcriptional factor, NF-κB, which in turn triggers expression of several pro- and anti-inflammatory genes^9^. However, immune cells are highly prone to oxidative stress induced by excessive amount of free radicals that can damage the signalling cascade. If the radicals are not neutralized by endogenous antioxidant system the consequence can lead to generation of several chronic diseases. In this backdrop, adequate amount of antioxidant supplementation is mandatory to prevent the damage^10^. Thus, there has been increasing interest to search for natural bioactive carbohydrate with safety and efficacy where traditionally prized mushrooms are regarded as an important bio-resource. Conversely, limited work has been performed on macrofungal polysaccharide isolated by alkaline solvent recycling remainder of hot water process^3,11^.

Therefore, the present study was devoted for preparation of carbohydrate rich fraction from *R*. *alatoreticula* following a unique extraction protocol to extend utilization of that novel taxon. The formulation was further investigated to determine physico-chemical characters and medicinal attributes in respect to antioxidant as well as immunostimulatory activities.

## Results and Discussion

### Physico-chemical characterization of RualaCap

**Figure 1:**
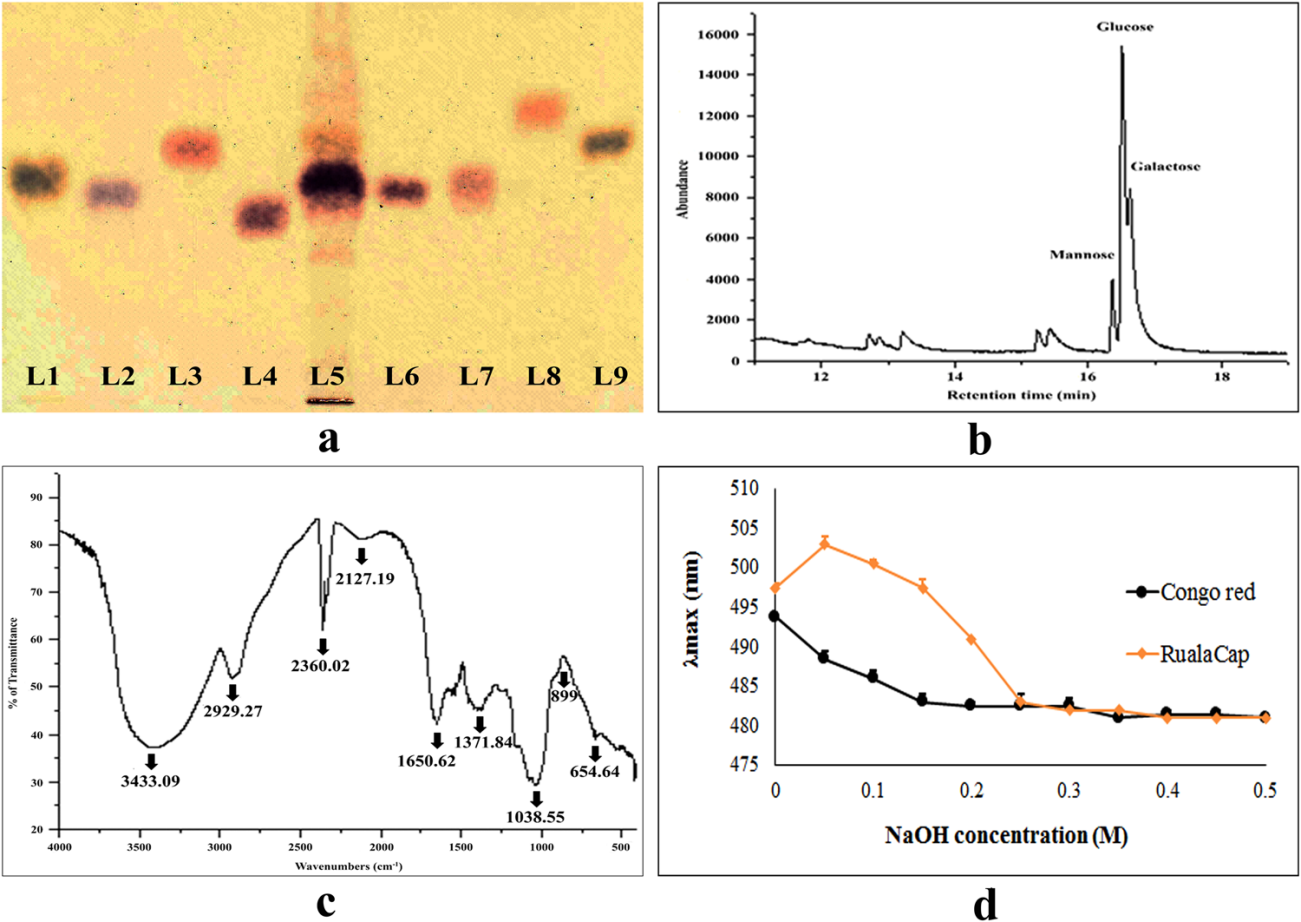
Structural and molecular characterization of cold alkaline extracted crude polysaccharide, RualaCap, isolated from *Russula alatoreticula*. (**a**) Identification of monosaccharides in hydrolysed polysaccharides by HPTLC, Lanes: 1: L-arabinose, 2: D-fructose, 3: D-fucose, 4: D-galactose, 5: RualaCap, 6: D-glucose, 7: D-mannose, 8: D-rhamnose, 9: D-xylose (**b**) GC-MS chromatogram of derivatized polysaccharide (Retention time of D-mannose: 16.6 min, D-glucose: 16.7 min, D-galactose: 16.8 min) (**c**) FT-IR spectrum (**d**) Changes in absorption maximum of Congo red-polysaccharide complex at various concentrations of sodium hydroxide solution.

To prepare polysaccharidic fraction from *R*. *alatoreticula* a quite distinct extraction procedure was followed that involved leftover residue of hot water process and alkali as extractant solvent. Finally, a water soluble fraction was isolated from alkali treated polysaccharide, designated as RualaCap, which appeared light brown in colour. As expected, the technique resulted in high recovery percentage which was about four times better than hydrothermal process^3^. Afterwards, the polymers were investigated chemically and the results have been listed in Table 1. Overall, carbohydrate was detected as the major constituent conjugated with small amount of protein. Besides, spectroscopic outcome implied that β-glucan was the dominant component of polysaccharide backbone while α-linked glucose was presented in trace. The data was further confirmed by high-performance thin-layer chromatography (HPTLC) where the chromatogram displayed highly intense band corresponding to D-glucose (Fig. 1a). However, gas chromatography–mass spectrometry (GC–MS) analysis revealed presence of three monosaccharides which were existed in the order of glucose > galactose > mannose (Fig. 1b).

**Table 1:** Chemical characterization of cold alkaline extracted crude polysaccharide, RualaCap from *Russula alatoreticula* (mean ± standard deviation; n = 3).

Parameters	RualaCap
Yield of polysaccharide (gm/100 gm of dry powder)	12 ± 1.63
Total carbohydrate (gm/100 gm of polysaccharide)	60.87 ± 8.39
Total glucan (gm/100 gm of polysaccharide)	42.68 ± 4.27
Total β-glucan (gm/100 gm of polysaccharide)	39.13 ± 4.13
Total α-glucan (gm/100 gm of polysaccharide)	3.55 ± 0.13
Total protein (gm/100 gm of polysaccharide)	6.46 ± 0.26
Monosaccharide composition	Mannose: Glucose: Galactose = 1: 3.83: 2.1

In general, structure of mushroom polysaccharides are highly diversified due to different sugar composition, linkage type, conformation, molecular weight, and so forth, which together execute significant effects on the bioactivity^12^. For instance, mannose was identified as an important component of the carbohydrates extracted from *Inonotus obliquus* and *Tremella mesenterica* displaying antioxidant and immune-enhancing properties respectively. Conversely galactose and glucose, detected as the active component in polysaccharide from *Agrocybe cylindracea*, played a major role behind antioxidant activity. In another study, free radical scavenging effect of *Hirsutella* sp was reported to be strongly dependent on mannose and galactose^13^. Besides D-glucose monomers linked by β-glycosidic bonds, called β-D-glucan, also have great potential in a wide array of fields due to their configuration and potent pharmacological properties^14^. Therefore, it could certainly be said that presence of these units in RualaCap might also be helpful to accomplish its functional property.

### Fourier-transform infrared spectroscopy (FT-IR) analysis

FT-IR spectroscopy is a simple, time saving, accurate and non-destructive technique that requires small amount of sample to predict functional groups^15^. As presented in Fig. 1c, a broad band of OH group was detected at 3433.09 cm^−1^ and a weak signal of C-H stretching vibration was noticed at 2929.27 cm^−1^. An asymmetrical stretching peak at 1650.62 cm^−1^ and a weaker symmetric stretching band near 1400 cm^−1^ were observed suggesting presence of uronic acid. Besides, the spectrum illustrated characteristic bands within 1200–1000 cm^−1^ that could be assigned to glycosidic linkage (C-C and C-O stretching vibration of pyranose ring). The signal of 899 cm^−1^ could be attributed to C-H band in β-configuration^16,17^. Based on aforementioned results, it could be assumed that RualaCap was a carbohydrate rich fraction consisting sugar units in β-type configuration.

### Depiction of helical conformation by Congo red reaction

Mushroom polysaccharides are known to exist in single or triple helical configurations, as well as random coil that can be detected by Congo red. This colorimetric assay is based on binding of the dye with helical polysaccharide resulting in bathochromic shift from 488 to 516 nm (>20 nm)^18^. Accordingly, the consequence of Congo red and RualaCap mixture was evaluated for conformational characterization. As presented in Fig. 1d, the reactant solution exhibited an initial 5 unit shift from 497 to 503 nm and then λmax decreased gradually. This could be explained by the steps of extraction process where NaOH was used that is responsible for breakage of helical form. So far, several bioactive polysaccharides are reported to be organised in triple helical structure. In contrast to that, significant immune-enhancing as well as antioxidant activities were exhibited by bio-polymers extracted from *Russula senecis*^3^, *Russula albonigra*^19^ and *Ganoderma lucidum*^4^ that did not contain any ordered structure.

### Evaluation of antioxidant activities of RualaCap

**Figure 2:**
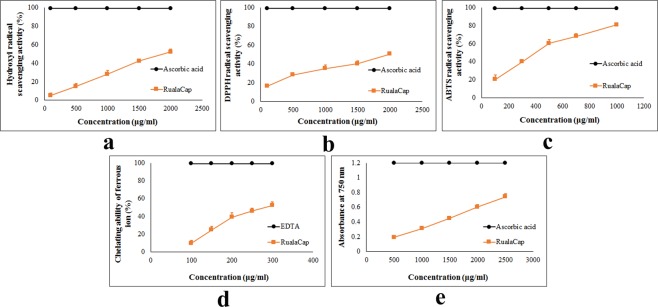
Antioxidant activity of cold alkaline extracted crude polysaccharide, RualaCap, prepared from *Russula alatoreticula*. (**a**) Hydroxyl radical scavenging activity (**b**) DPPH radical scavenging activity (**c**) ABTS radical scavenging activity (**d**) Chelating ability of ferrous ion (**e**) Reducing power.

Antioxidative compounds are saviour of cellular damage from oxidative stress as they are able to donate hydrogen or electron and quench free radicals^20^. Thus in search of new biomaterials with direct antioxidant ability, RualaCap was subjected to six *in vitro* systems and the outcome has been summarized in Table 2. Initially, the assay of hydroxyl radical scavenging activity was performed as this highly reactive radical is capable of destroying almost every cellular molecule. Results showed that the extract inhibited generation of 5.74%, 14.99%, 28.07%, 42.22% and 52.14% radical at the level of 100, 500, 1000, 1500 and 2000 μg/ml respectively (Fig. 2a). The similar trend of radical quenching ability was also observed in case of 2,2-diphenyl-1-picrylhydrazyl (DPPH), a commercially available organic compound that has widely been used to evaluate antioxidant activity of investigating drug. Eventually, RualaCap displayed a significant concentration-wise action pattern and results were plotted in Fig. 2b. At level of 100, 500, 1000, 1500 and 2000 μg/ml of extract the quenching rate was 16.11%, 28.33%, 35.12%, 40.26% and 50.5% respectively demonstrating adequate ability. In addition, 2, 2′-azino-bis (3-ethylbenzothiazoline-6-sulphonic acid) (ABTS) was also used in the present study to further assess antioxidant effect. Results depicted that the polysaccharide possessed strong radical scavenging capacity in a concentration dependent manner (Fig. 2c). As the level ranged from 100, 300, 500, 700 to 1000 μg/ml, inhibition activities of RualaCap amplified from 20.44%, 40.15%, 60.34%, 68.12% to 80.92% respectively. Moreover, Fe^+2^ chelating activity of the extract was determined as well due to involvement of this metal in formation of radicals inside human body. As presented in Fig. 2d, the chelating ability of polysaccharides was 10.11%, 25.48%, 39.09%, 46.72% and 52.27% at the level of 100, 150, 200, 250 and 300 μg/ml respectively depicting strong binding affinity towards the ion. Further, ferricyanide/prussian blue assay was carried out to determine reducing power of RualaCap where the polysaccharides exhibited moderate ability to convert Fe^+3^ to Fe^+2^ (Fig. 2e). At the concentrations of 500, 1000 and 1500 µg/ml reducing power were 0.19, 0.31 and 0.45 that gradually elevated to 0.6 and 0.74 at the doses of 2000 and 2500 µg/ml. Finally, total antioxidant activity was determined and result showed that reducing capacity of 1 mg of the extract was equivalent to 1.47 µg of ascorbic acid.

**Table 2:** Antioxidant activity of cold alkaline extracted crude polysaccharide, RualaCap, isolated from *Russula alatoreticula*.

	Antioxidant Assays	RualaCap	Standard
EC_50_ value (µg/ml)	Scavenging ability of hydroxyl radicals	1848 ± 20^a^	69 ± 1^b^
	Scavenging ability of DPPH radicals	1976 ± 65^a^	4.5 ± 0.5^b^
	Scavenging ability of ABTS radicals	390 ± 9^a^	2.58 ± 0.09^b^
	Chelating ability of ferrous ion	283 ± 2^a^	2.54 ± 0.5^b^
	Reducing power	1687 ± 56^a^	14.5 ± 5^b^
Total antioxidant activity by phosphomolybdenum method (µg ascorbic acid equivalent/mg of dry polysaccharide)		1475 ± 2	NA

The results are presented in EC_50_ values (mean ± standard deviation; n = 3) corresponding to 50% of antioxidant activity or 0.5 of absorbance. Ascorbic acid was considered as a  standard in hydroxyl radicals scavenging, ABTS radical inhibition, DPPH radical quenching, reducing power and total antioxidant capacity techniques. While EDTA was adopted as a positive control in chelating ability of ferrous ion method. In each row, different letters mean significant differences between sample and standard (*p* < 0.05).

NA: Not applicable.

Till date, numerous studies have demonstrated the *in vitro* antioxidant activity exerted by polysaccharide from wild edible mushrooms. In contrast to that, limited information is available on bioactivity of *Russula* sp that comprise one of the largest and major ectomycorrhizal group distributed all over the world. However, comparative study with available evidences indicated that the studied fraction from *R*. *alatoreticula* exhibited better reducing power and chelating effect than the crude as well as pure polysaccharide from *Russula virescens*^21^. Besides, RualaCap also exhibited enhanced DPPH and ABTS radical scavenging properties than pure polysaccharide from *Russula griseocarnosa*^22^. Recently, Liu *et al*.^23^ have reported antioxidant activity of water and alkali-soluble polysaccharides from *Russula vinosa* and the outcome presented its poor hydroxyl radical scavenging, reducing power and chelating effect than RualaCap. In addition, the extract under investigation was also proved to possess better reducing power and ABTS radical quenching effect than alkaline extracted crude polysaccharide from *R*. *senecis*^3^. In contrast to that, the fraction presented lesser potency than pure polysaccharide from *R*. *albonigra* except chelating ability assay^19^. Moreover, hot water extracted polysaccharide from *R*. *alatoreticula*, Rusalan, was found to execute higher antioxidant property than its alkaline treated polysaccharide fraction, RualaCap^1^.

### Determination of immunostimulatory activity of RualaCap

#### Effect on RAW 264.7 cell proliferation and phagocytosis

**Figure 3:**
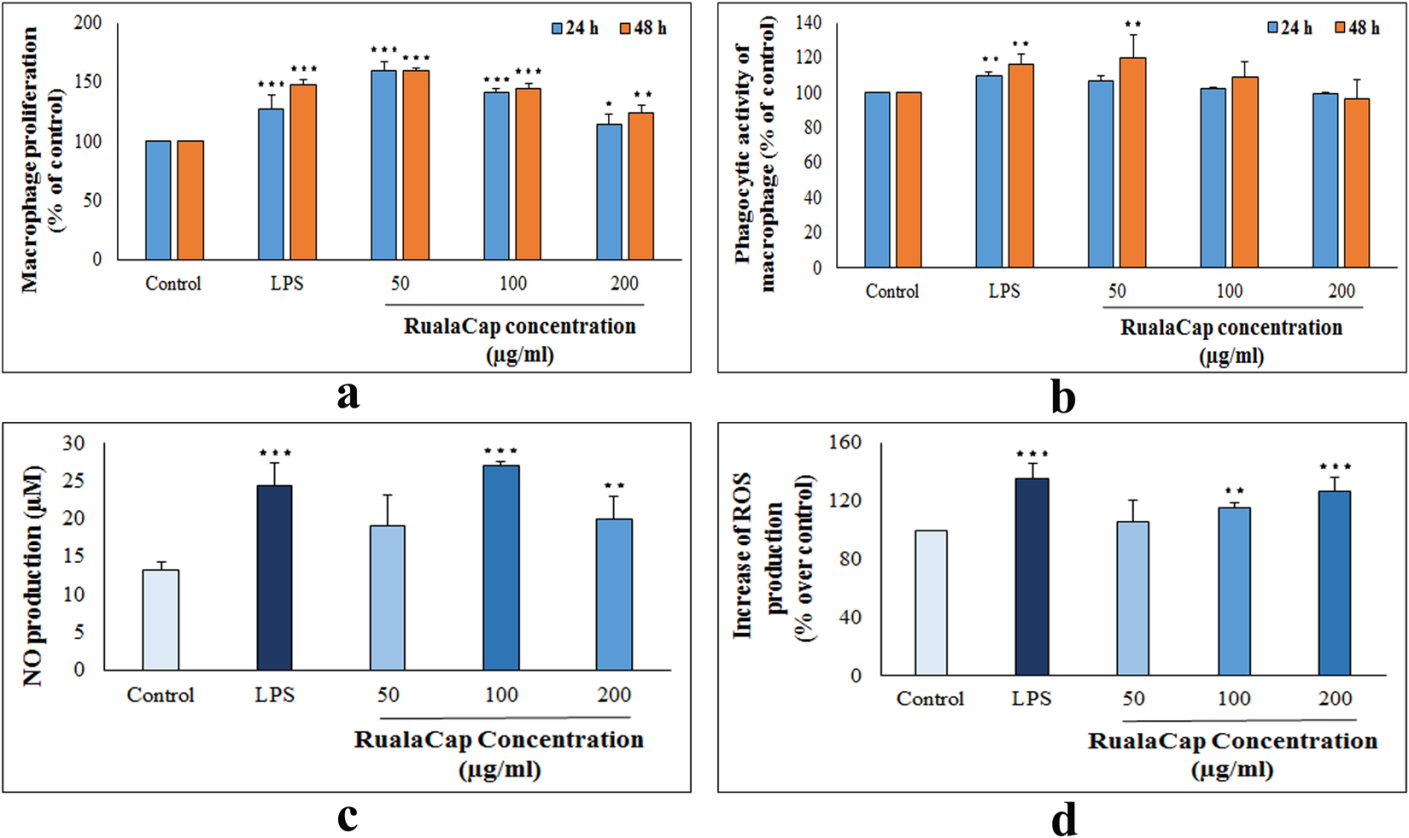
Effect of cold alkaline extracted crude polysaccharide, RualaCap, isolated from *Russula alatoreticula* on activity of macrophages. (**a**) Proliferation was monitored using water soluble tetrazolium (WST) in treatment of polysaccharide at different concentrations and time and expressed in relation (%) to negative control. (**b**) Phagocytosis in relation (%) to control was determined by neutral red method. (**c**) Release of NO in cell supernatant was quantified using Griess reagent. (**d**) Intracellular ROS generation was determined by flow cytometry (See also Supplementary Fig. S2). In all assays. LPS at the concentration of 5 µg/ml was used as a positive control. Results represent the mean ± standard deviation of at least three independent experiments. ANOVA *p* < 0.05; as regards Tukey’s *post-hoc* test the sign ‘*’ indicates significant differences compared to untreated control group. *p < 0.05, **p < 0.01, ***p < 0.001.

Macrophages are the key participant in innate immunity as they phagocyte non-self particles and secrete major modulators that activate adaptive response. Increase in their number and engulfment power can thus positively contribute more amounts of mediators and destruction of pathogens resulting pronounced activation of host defence mechanism^8,24^. Therefore to determine immune boosting effect, consequence of RualaCap on murine macrophage cell, RAW 264.7, viability and phagocytic uptake was assessed. As expected, the polysaccharide enhanced both the activities where the potency was even higher than standard, lipopolysaccharide (LPS). The fraction executed its effect within 24 h treatment where it showed the maximum function at only 50 μg/ml concentration. After 48 h, the proliferation frequency was incremented by 159.83%, 144.65% and 124.22% in treatment of 50, 100 and 200 μg/ml of RualaCap respectively (Fig. 3a). While, the phagocytosis was increased by 120.02%, 108.88% and 96.94% at those tested levels (Fig. 3b) (see also Supplementary Fig. S1). Literature survey implied that, the studied fraction exhibited better proliferation activity than pure polysaccharide from *R*. *griseocarnosa*^25^ and lower phagocytic effect than hot water extracted carbohydrate fraction from *R*. *alatoreticula*^1^.

#### Effect on NO production and intracellular ROS synthesis

During phagocytosis, ROS in form of superoxide radical is produced as part of oxidative burst that helps in eradication of intracellular parasites. The second oxidant synthesized by macrophages is NO which owe microbicidal potency by direct attack on bacteria, viruses and protozoa. In murine systems, NO is produced by iNOS activated via synergistic effect of IFN-γ and TNF^26^. Thus, increase in production of these multifunctional molecules can be one of the feasible target of immunemodulation in macrophages. Interestingly, RualaCap stimulated enhanced production of both NO and ROS within 24 h treatment. Results suggested that at concentrations of 50, 100 and 200 µg/ml, the polysaccharide induced 19.16, 27.04 and 20.07 µM NO generation respectively (Fig. 3c). While, the fraction at those doses elevated ROS synthesis by 105.84%, 114.99% and 126.54% in respect to untreated cells (Fig. 3d) (see also Supplementary Fig. S2). Consequently, the studied fraction presented better activity in these aspects than alkaline extracted crude polysaccharide from *R*. *senecis*^3^.

#### Detection of morphological changes of macrophages

**Figure 4:**
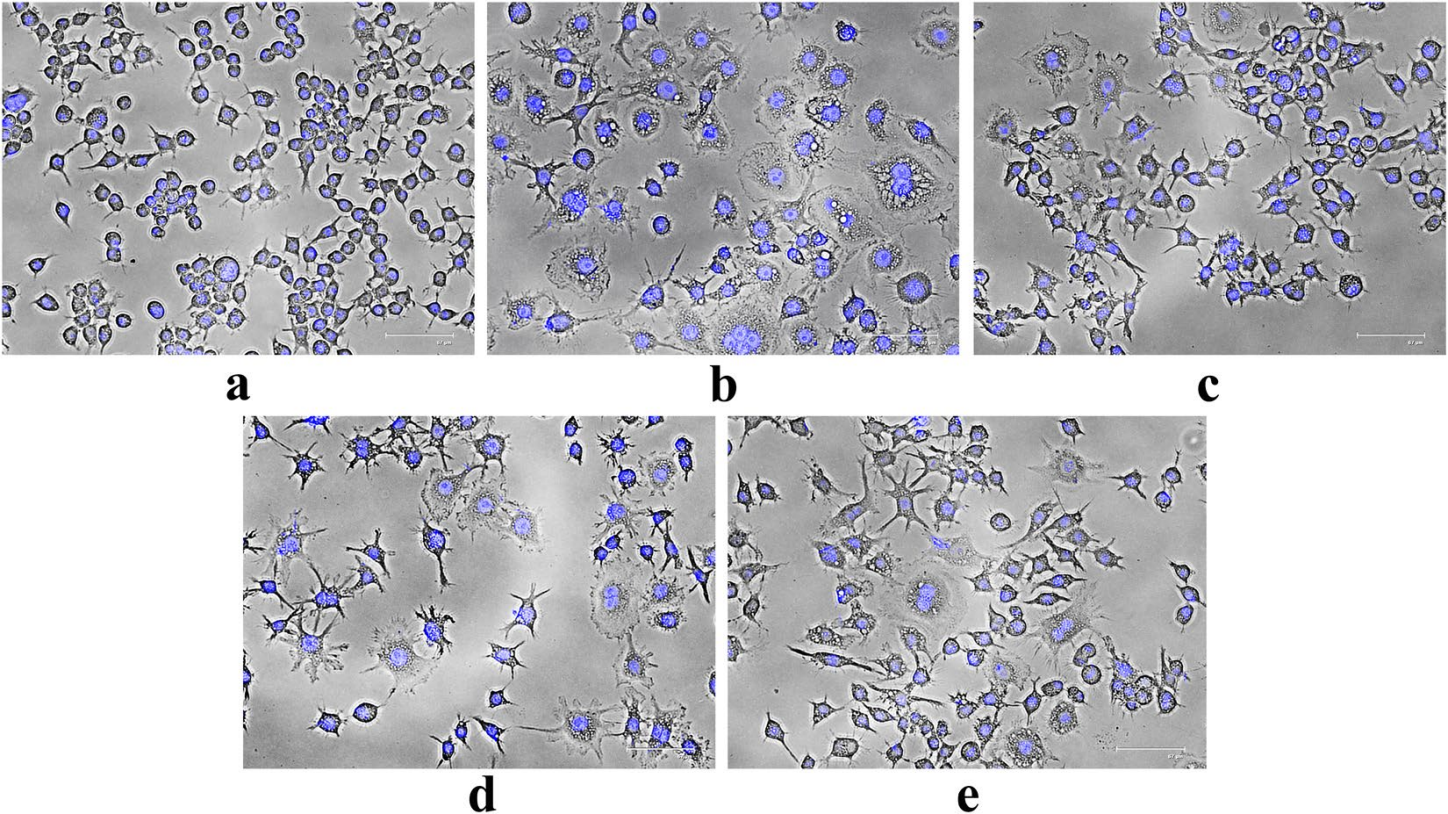
Effect of cold alkaline extracted crude polysaccharide, RualaCap, isolated from *Russula alatoreticula* on morphology of macrophages. Cells were incubated for 24 h with different concentrations of extract where LPS at the level of 5 µg/ml was used as a positive control. Afterwards cells were fixed, subjected to fluorescence microscopy and images were captured. (**a**) Negative control (**b**) LPS (c) 50 µg/ml (**d**) 100 µg/ml (**e**) 200 µg/ml.

When activated, morphology of macrophage cell changes from generic round shape to more specific dendritic structure. Such alteration facilitates them to become antigen presenting cells for naive T lymphocytes during primary immune response^27^. These activated monocytes are then characterized by a number of thin sheets from cell edges, called filopodia or lamellipodia^28^. Thus to investigate effect of RualaCap on macrophage architecture, cells were treated with the fraction at various doses. As presented in Fig. 4, the polysaccharides as well as LPS induced morphodynamics in RAW 264.7 cells including remarkable increase in cell size, number and spreading in contrast to untreated set. In addition, RualaCap also stimulated production of numerous hair like membrane protrusions and the effect was more pronounced in treatment of 100 μg/ml concentration. The apparent results were found to be in accordance with our previous works^1,3^.

#### Measurement of gene expression and elucidation of mode of action

Over the past decade, a large number of studies have revealed that TLRs play important roles in host defence and among the different types TLR-2 as well as TLR-4 are the best characterized. After binding with specific ligands, both TLRs drive downstream signalling cascade that involves stimulation of a common set of proteins, the most significant of which is activation of NF-κB^29^. NF-κB then binds to its consensus promoter DNA sequences and triggers expression of target genes such as TNF-α, iNOS, COX-2, Iκ-Bα, IFN-γ and so forth^30–35^. TNF-α is one of the early pro-inflammatory cytokine synthesized by macrophages which in turn induces production of other mediators. Conversely, expression of iNOS results in production of NO, as mentioned before, and overproduction of NO further stimulates synthesis of COX-2^36^. However, persistent release of pro-inflammatory cytokines causes deleterious effect and thus production of anti-inflammatory modulator such as IL-10 is essential during immune response^8^.

**Figure 5:**
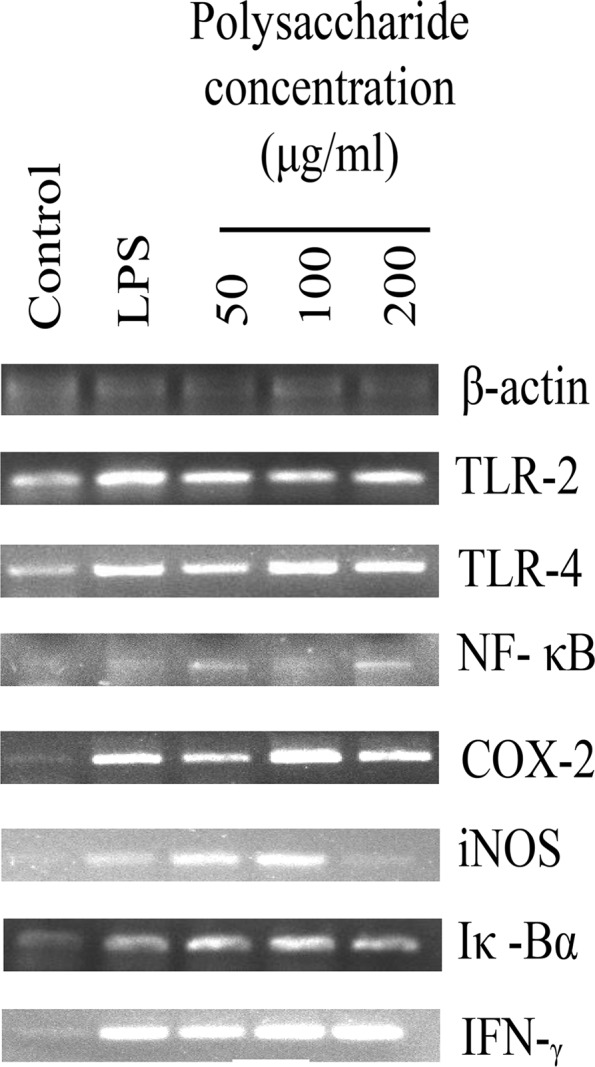
Effect of cold alkaline extracted crude polysaccharide, RualaCap, prepared from *Russula alatoreticula* on mRNA expression. Total RNA was isolated from macrophage cells after 24 h incubation either with LPS (5 µg/ml concentration) or RualaCap (50, 100 and 200 µg/ml concentration) along with untreated cells. cDNA was prepared from respective RNA samples and semi-quantitative reverse transcriptase PCR was performed to analyse the expression of seven different genes such as TLR-2, TLR-4, NF-κB, COX-2, iNOS, Iκ-Bα and IFN-γ where β-Actin was considered as a house keeping gene. Full-length gel pictures are presented in Supplementary Fig. S4.

**Figure 6:**
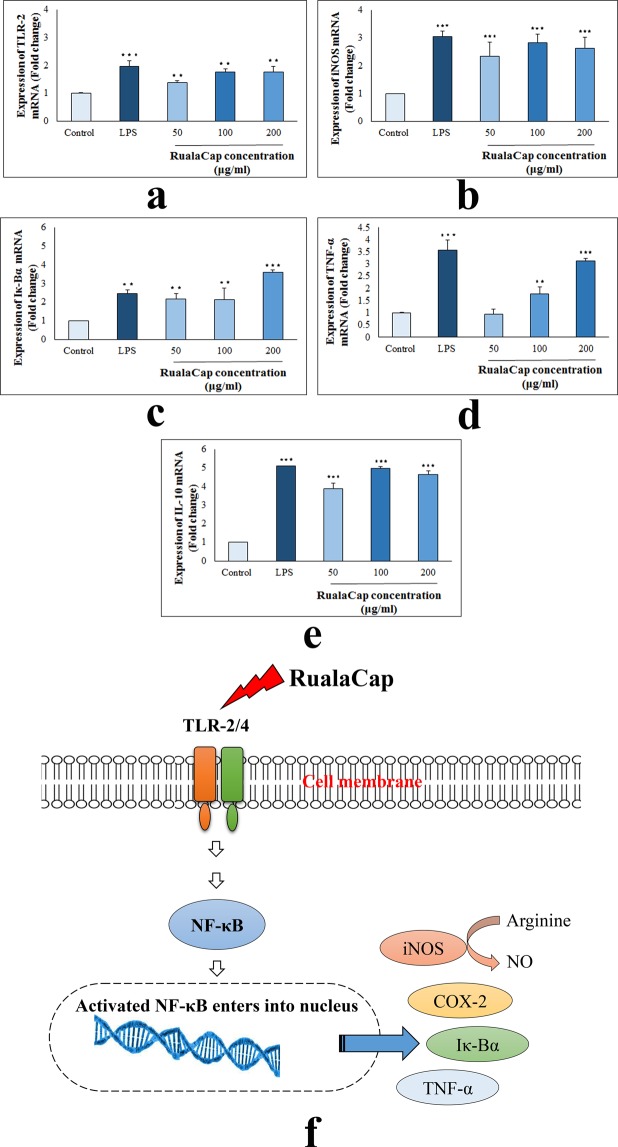
Analysis of mechanism of action by cold alkaline extracted crude polysaccharide, RualaCap, prepared from *Russula alatoreticula.* Real time PCR was implemented to detect expression level of five genes quantitatively (**a**) TLR-2 (**b**) iNOS (**c**) Iκ-Bα (**d**) TNF-α (**e**) IL-10. Values were represented as mean ± standard deviation of at least three independent experiments. ANOVA *p* < 0.05; as regards Tukey’s *post-hoc* test the sign “*” indicates significant differences compared to untreated control group. **p < 0.01, ***p < 0.001. (**f**) The schematic diagram represents immune stimulatory activity of RualaCap mediated through TLR/NF-κB pathway. The polysaccharide fraction being enriched in β-glucan acts as ligand for TLR2 and TLR4 and consequently activates downstream signalling pathway. As a result the transcription factor, NF-κB, becomes stimulated that subsequently binds to promoter region of a number of genes resulting synthesis of important mediators. These mediators play a significant role for activation of adaptive immune response and thus enhance overall host defence mechanism.

Therefore to determine whether RualaCap executed its function thorough these pro- and anti-inflammatory modulators, mRNA expressions of total nine genes were studied. Results showed that after incubation with the polysaccharides for 24 h, transcription of all investigating genes was significantly elevated compared to negative control (Fig. 5) (see also Supplementary Fig. S3). The outcome was further verified by qRT-PCR and overall transcriptome analysis indicated that the extract at 100 μg/ml concentration possessed the best stimulatory effect. At that dose, RualaCap induced expressions of TLR-2, iNOS, Iκ-Bα, TNF- α and IL-10 by 1.77, 2.83, 2.14, 1.77 and 4.96 fold respectively (Fig. 6a–e). Interestingly, the overall effect was found to be highly comparable with LPS portraying effective immune boosting potential of RualaCap.

Since antiquity, many higher fungi of Basidiomycetes have been used in ethnic medicines owing to immune potentiation that has nowadays attributed to β-glucan. Fungal β-glucan, in contrast to α-linked glucan, are not digested by human enzymes when orally administered. Instead they are taken up in small intestine and stimulate systematic immune response^37^. Although the mechanism has at present begun to be elucidated, research till date have illustrated that β-glucan is recognized by macrophage receptors like TLR-2/4 and induce signalling cascade^38^. However, scarce information is available explaining mode of action behind immunostimulatory activity of many traditionally opted mushrooms including *Russula* sp. Recently, Chen *et al*.^25^ have showed that purified polysaccharide from *R*. *griseocarnosa* regulated immune activity via NF-κB and MAPK pathways. Besides in our previous works, *R*. *alatoreticula*^1^ and *R*. *senecis*^3^ exhibited their immunologic functions facilitated by several cytokines; although the underlying pathway remain anonymous. Thus, the present study might be considered important as it shed light on mechanistic insight of the studied folk mushroom (Fig. 6f).

In summary, we present a bioactive fraction, RualaCap, isolated by using residue of hot water process to broaden application of ethnically prized myco-food, *R*. *alatoreticula*. The crude polysaccharide executed effective antioxidant potential where EC_50_ values were in order of chelating ability of Fe^2+^ > ABTS radical scavenging > reducing power > hydroxyl radical inhibition > DPPH^.^ quenching activity. In addition, notable immune-boosting effect was also observed as reflected by augmentation in cell division, engulfment power, filopodia formation and level of pro- (TNF-α, COX-2, IFN-γ, iNOS, NO, ROS) as well as anti-inflammatory (IL-10) mediators by macrophages. Detailed study illustrated that the function was mediated thorough TLR2 and TLR4 activated NF-κB pathway. Such therapeutic potentiation could be explained by presence of β-glucan detected as prominent component in the fraction. Thus, the results may lead to further development of RualaCap as an agent to improve oxidative stress induced ailment and integrity of our immune system, especially against immune-deficiency condition.

## Materials and Methods

### Cell line and chemicals

RAW 264.7 murine macrophages were purchased from National Centre for Cell Science, Pune, India. 2-Deoxy-D-ribose, NaOH, ferric chloride, hydrogen peroxide, thiobarbituric acid, ferrozine, trichloroacetic acid, potassium ferricyanide, trifluoroacetic acid, DPPH, ABTS, sodium persulfate, ammonium molybdate, sodium borohydride, pyridine, ascorbic acid, ethylenediaminetetraacetic acid (EDTA), bovine serum albumin (BSA), LPS and monosaccharides were procured from Sigma chemicals Co. (St. Louis, MO, USA). A mushroom β glucan kit was used from Megazyme Institute Wicklow, Ireland. Dulbecco’s Modified Eagle Medium (DMEM), neutral red, sulfanilamide, naphthylethylenediamine dihydrochloride, phosphoric acid, Congo red, 2′,7′-dichlorofluorescin diacetate (DCFDA), 4′,6-diamidino-2-phenylindole (DAPI) were purchased from Himedia, Mumbai, India. WST was obtained from Takara Bio Inc, Japan. Fetal bovine serum (FBS) and TRIzol were purchased from Invitrogen, New Delhi, India. PenStrep, amphotericin B and cDNA preparation kit were used from MP Biomedicals, Santa Ana, CA, USA. PowerUp^TM^ SYBR^®^ Green Master Mix was procured from Applied Biosystems, USA.

### Collection and authentication

Basidiocarps of *R*. *alatoreticula* were collected from natural habitat of West Bengal in the month of July. Identity of the collected basidiome was confirmed based on morphological and DNA barcoding analyses as described in our previous publication^1^.

### Extraction of crude polysaccharide (RualaCap)

Dried and powdered fruit bodies were first refluxed with ethanol to eliminate the alcohol soluble components. The air dried filtrate was then submerged in distilled water at boiling condition for 7 h. The residue was separated using nylon cloth and then subjected to 10% NaOH solution at 4 °C for overnight. The resultant supernatant was neutralized by glacial acetic acid and precipitated by addition of four volume of absolute alcohol. After centrifugation (11,000 rpm for 10 min at 4 °C), pellets were collected and dissolved in water repeatedly to isolate the water soluble fraction. The extract was concentrated to one tenth volume and alcohol precipitated pellets were recovered by centrifugation followed by stepwise washing with ethanol and acetone^3,11^. Finally, it was kept in amber containers under dry condition to yield the water soluble fraction of cold alkaline extracted polysaccharide from *R*. *alatoreticula* designated as RualaCap.

### Determination of architecture of RualaCap

Total sugar content was measured by phenol sulphuric acid method using glucose as standard and results were expressed as gm of glucose equivalent/100 gm of dry polysaccharide. Quantity of total glucan and its types were estimated using mushroom and yeast β-glucan assay kit as per the manual. All values of glucan contained were expressed as gm of glucose equivalent/100 gm of dry polysaccharide. Protein concentration was determined using the method described by Bradford and expressed as gm of BSA equivalent/100 gm of dry polysaccharide. Further, RualaCap was subjected to molecular composition analysis by HPTLC and GC-MS^11^. FT-IR spectrum was recorded on PerkinElmer Precisely Spectrum 100 Model (USA) in frequency range 400–4000 cm^−1^. Finally, helical structure of carbohydrate backbone was analysed by characterizing Congo red-polysaccharide reaction according to the method described by He *et al*.^39^.

### Evaluation of antioxidant activities of RualaCap *in vitro*

The technique proposed by Halliwell *et al*.^40^ was followed for determination of hydroxyl radical scavenging activity. The radicals were produced in 1 ml solution by Fenton’s reaction containing variable concentrations (100–2000 μg/ml) of RualaCap and absorbance was measured at 535 nm. In addition, the antioxidant activity was also evaluated using DPPH^.^ adopting 96 well plate. DMSO solution of the radical (0.1 mM) was evaluated against various levels of crude polysaccharide (100–2000 μg/ml) and absorbance was detected at 595 nm using microplate reader (Bio-Rad iMark^TM^ Microplate Reader, USA). Further, potential against ABTS radical was evaluated using (100–1000 μg/ml) concentrated extract and absorbance was detected at 750 nm. Moreover, the ability of investigated fraction to chelate ferrous ion was determined as well with different level of RualaCap (100–300 μg/ml). Nevertheless, a modified method of reducing power was considered also where variable concentrations of polysaccharides (500–2500 μg/ml) were mixed in microtiter plate and the absorbance was measured at 750 nm^41^. Finally, the assay of total antioxidant capacity was carried out as described by Prieto *et al*.^42^ and activity was expressed as number of equivalent of ascorbic acid.

### Determination of immunostimulatory activity of RualaCap

#### Cell Culture

RAW 264.7 cells were maintained in DMEM supplemented with 10% (v/v) FBS, 0.5% (v/v) PenStrep (5,000 IU/ml penicillin and 5 mg/ml streptomycin) and 0.25% (v/v) amphotericin B (250 µg/ml). In all sets, LPS at 5 µg/ml concentration was used as a positive control; while RualaCap at the level of 50, 100 and 200 µg/ml were considered as experimental doses.

#### Cell proliferation assay

Different concentrations of RualaCap were added to monolayer culture of macrophage cells (3000 cells/well) in 96 well culture plate and incubated. After 24 and 48 h treatment, the cell viability was determined by WST reagent and absorbance was measured at wavelength of 450 nm.

#### Phagocytosis assay

After macrophage activation with different concentrations of RualaCap for 24 and 48 h, reaction mixture was discarded and incubated with 100 μl DMEM containing neutral red followed by washing the cells with PBS twice. Then 100 μl of cell lysate reagent was added into each well to lyse cells and optical density was measured at 575 nm using microplate reader^1,3^.

#### Determination of NO and ROS production

Following 24 h treatment, culture supernatant was mixed with an equal volume of freshly prepared Griess reagent and absorbance was measured at 545 nm. The nitrite concentration was determined by extrapolation based on a standard sodium nitrite (1–200 μM in culture medium) curve^1,3^. While, the cells were harvested and incubated with ROS indicator, DCFDA, at 37 °C for 30 min^43,44^. The intracellular ROS levels were measured with flow cytometry (BD Bioscience, USA) and analysed by BD CellQuest Pro software.

#### Detection of morphological changes of macrophages

RAW cells were cultured with various concentrations of RualaCap and allowed for incubation of 24 h. The adherent cells were fixed with methanol for 10 min, washed with PBS and incubated with DAPI solution for 15 min in dark. Cellular morphology was viewed and photographed using fluorescent microscope (FLoid Cell Imaging Station, Life Technologies, India).

#### RNA extraction, RT-PCR

The total RNA of macrophage cells after 24 h treatment was extracted and reverse transcribed into cDNA. The cDNA was further amplified using primers specific to TLR-4, TLR-2, NF-κB, Iκ-Bα, COX-2, iNOS, TNF-α and IFN-γ genes where β-actin was used as control. The PCR cycle conditions were as follows: 95 °C for 4 min, then 35 cycles of 94 °C for 20 s, annealing temperature (Tm) for specific primer (mentioned in Table 3) for 30 s and 72 °C for 45 s with a final extension step of 7 min at 72 °C in a thermal cycler (Applied BioSystem, USA).

**Table 3:** Mouse specific RT-PCR and qRT-PCR primer sequences.

Sl no.	Primer name	Primer sequence	Tm (°C)	Product size (bp)	Reference
1	TLR-4	F5′ CAGCTTCAATGGTGCCATCA 3′	54	399	^30^
		R5′ CTGCAATCAAGAGTGCTGAG 3′			
2	TLR-2	F5′ CACCACTGCCCGTAGATGAAG 3′	57	147	^31^
		R5′ AGGGTACAGTCGTCGAACTCT 3′			
3	NF-κB	F5′ AGAAGGCTGGGGTCAATCTT 3′	51	215	^32^
		R5′ CTCAGGCTTTGTAGCCAAGG 3′			
4	Iκ-Bα	F5′ CTTGGTGACTTTGGGTGCTGAT 3′	57	100	^33^
		R5′ GCGAAACCAGGTCAGGATTC 3′			
5	iNOS	F5′ GAGCGAGTTGTGGATTGTC 3′	55	327	^34^
		R5′ GGGAGGAGCTGATGGAGT 3′			
6	COX-2	F5′ CCCCCACAGTCAAAGACACT 3′	57	469	^35^
		R5′ GAGTCCATGTTCCAGGAGGA 3′			
7	IFN-γ	F5′ CCTCAAACTTGGCAATACTCA 3′	54	227	^32^
		R5′ CTCAAGTGGCATAGATGTGGA 3′			
8	TNF-α	F5′ GGGGATTATGGCTCAGGGTC 3′	55	149	^32^
		R5′ CGAGGCTCCAGTGAATTCGG 3′			
9	IL-10	F5′ TACCTGGTAGAAGTGATGCC 3′	56	251	^35^
		R5′ CATCATGTATGCTTCTATGC 3′			
10	β-Actin	F5′ GCTGTCCCTGTATGCCTCT 3′	55	221	^34^
		R5′ TTGATGTCACGCACGATTT 3′			

#### qRT-PCR

The mRNA expression of TLR-2, Iκ-Bα, iNOS, TNF-α and IL-10 genes were carried out with CFX 96^TM^ Real-Tis°C for 2 min, followed by 40 cycles of 95 °C for 15 s, 55 °C for 15 s and 72 °C for 30 s. The relative expression level was calculated following 2^−∆∆^Ct method where CT indicates cycle threshold^45^. All the genes were assayed in triplicate and mean expression values were used.

#### Statistical analysis

IBM SPSS Statistics for Windows, version 23.0. (IBM Corp., Armonk, NY, USA) was used as statistical analysis software. Experiments were performed at least in triplicate and results are presented as mean ± standard deviation. One-way analysis of variance (ANOVA) followed by Tukey’s *post-hoc* test was employed to assess significant differences (*p* < 0.05).

## Supplementary information

Supplementary material

## Data Availability

The data associated with a paper is available.
