# Supplementary material for: Alkali treated antioxidative crude polysaccharide from Russula alatoreticula potentiates murine macrophages by tunning TLR/NF-κB pathway
Source: Sci Rep. 2019 Feb 8;9:1713. doi: 10.1038/s41598-018-37998-2 (PMC6368593; doi:10.1038/s41598-018-37998-2)
Supplement: Supplementary file 1 — Supplementary material [file 41598_2018_37998_MOESM1_ESM.pdf]

Supplementary material

Alkali treated antioxidative crude polysaccharide from *Russula alatoreticula* potentiates murine macrophages by tunning TLR/NF-κB pathway

Somanjana Khatua, Krishnendu Acharya*

Molecular and Applied Mycology and Plant Pathology Laboratory, Centre of Advanced Study, Department of Botany, University of Calcutta, 35, Ballygunge Circular Road, Kolkata– 700019, West Bengal, India.

* To whom correspondence should be addressed: krish_paper@yahoo.com

Figure S1. Effect of cold alkaline extracted crude polysaccharide, RualaCap, isolated from *Russula alatoreticula* on phagocytic uptake of macrophages. Yeast cell suspension was prepared by mixing 30 mg of *Saccharomyces cerevisiae* in 10 ml of PBS and then cells were heat killed at 80°C for 15 min. Finally, 100 µl from 1×10^8 cells/ml suspension were flooded on to macrophages that were treated with polysaccharide or LPS for 24 h. After incubation at 37°C for 2 h, monocytes were washed with PBS, stained with Giemsa and observed under microscope. (**a**) Untreated cells (**b**) LPS at 5 µg/ml (**c**) RualaCap at 100 µg/ml.

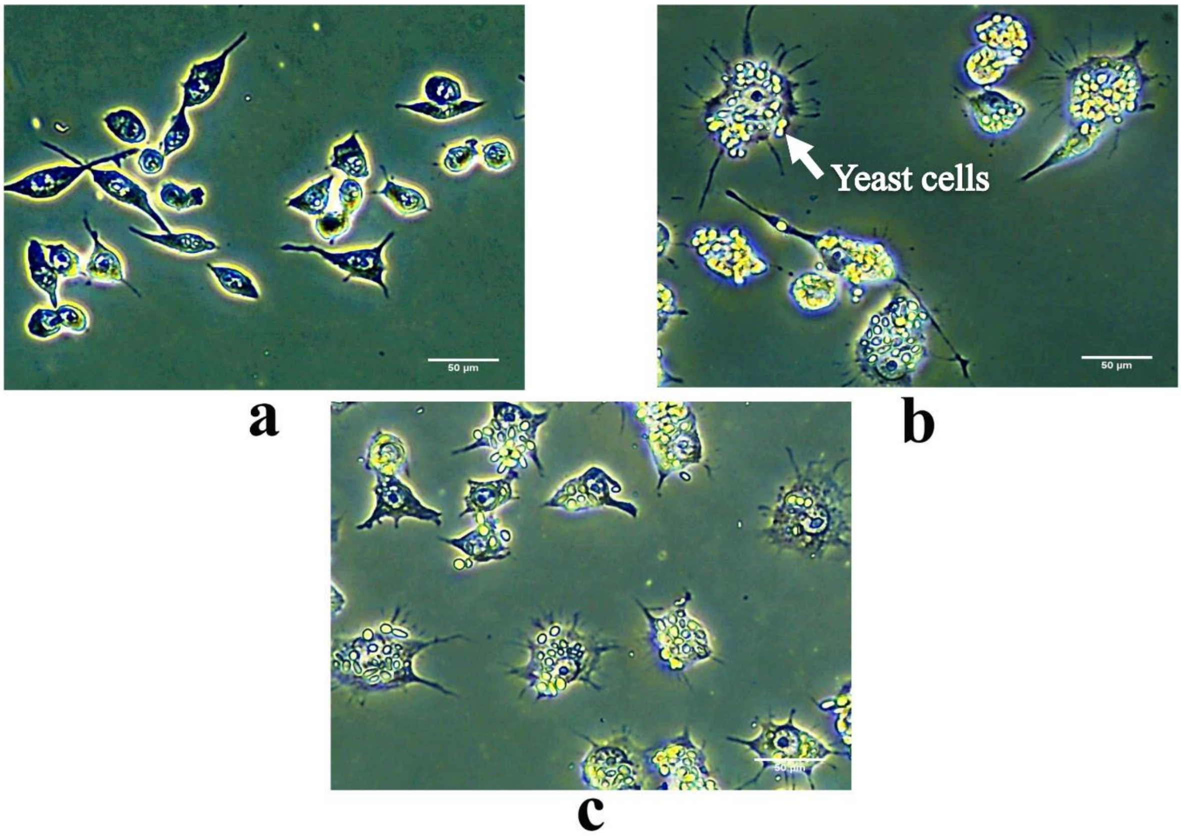

Figure S2. Effect of cold alkaline extracted crude polysaccharide, RualaCap, isolated from *Russula alatoreticula* on ROS production by macrophages. After 24 h incubation, intracellular ROS generation was determined by flow cytometry using DCFDA dye. Red coloured graphs represent log fluorescence intensity of oxidative product of DCFDA treated with (**a**) LPS at 5 µg/ml concentration and RualaCap at variable doses such as (**b**) 50 (**c**) 100 as well as (**d**) 200 µg/ml in comparison with negative control denoted by black coloured graph.

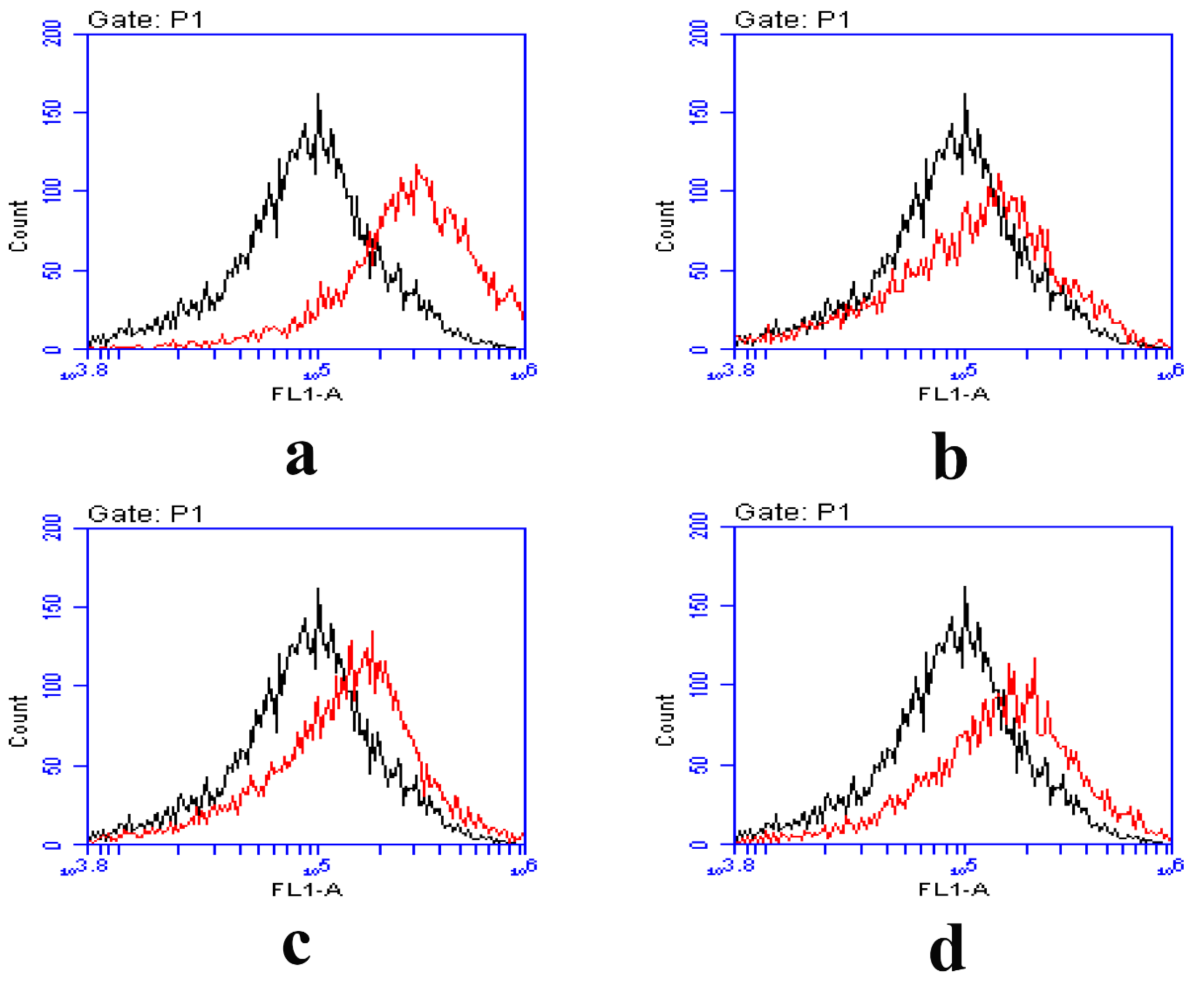

Figure S3. Gradient PCR was performed to standardize melting temperature (Tm) for primers and determine amplicon size. Lane 1: GAPDH (56ºC), Lane 2: iNOS (53ºC), Lane 3: iNOS (55ºC), Lane 4: IL-6 (51ºC), Lane 5: IL-10 (56ºC), Lane 6: IL-10 (59ºC), Lane 7: COX-2 (53ºC), Lane 8: COX-2 (57ºC), Lane 9: TNF-α (55ºC), Lane 10: TNF-α (56ºC), Lane 11: β-actin (55ºC), Lane 12: β-actin (57ºC), Lane 13: DNA Ladder (100-1000 bp)

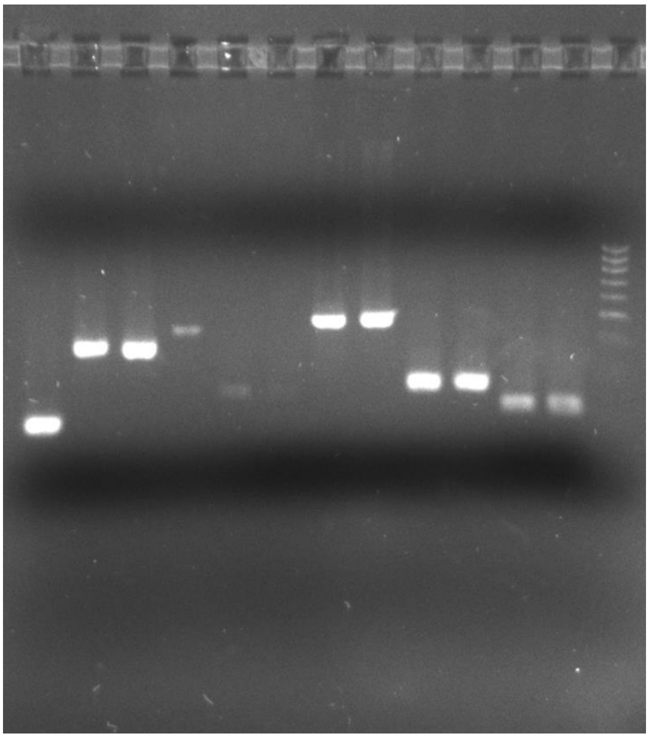

Figure S4. Full length gel pictures of genes prepared by reverse transcriptase PCR as presented in Figure 5

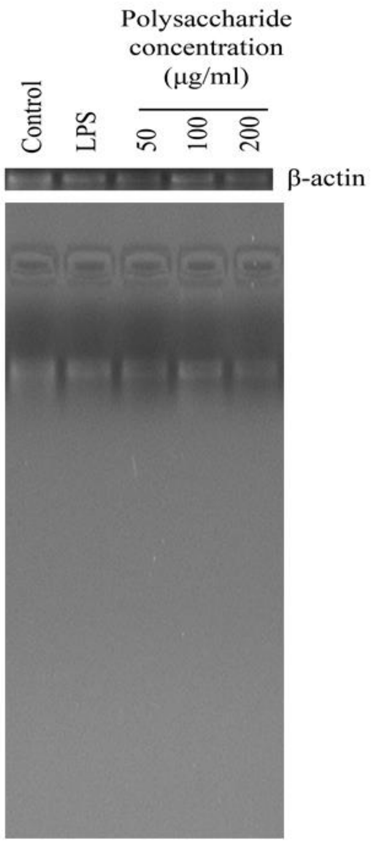
Polysaccharide
concentration
(μg/ml)
Control
LPS
50
100
200
β-actin

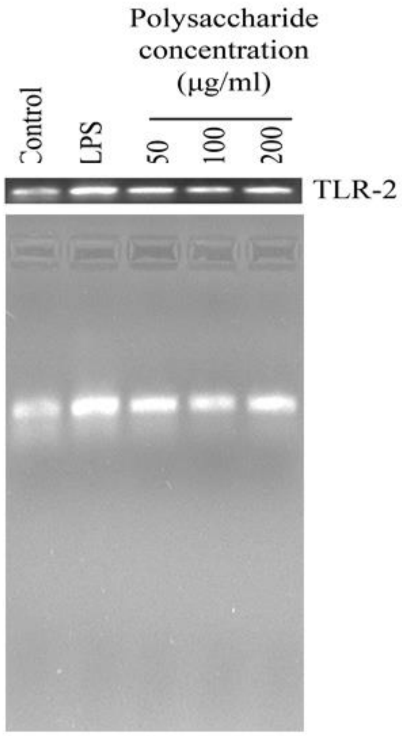
Polysaccharide
concentration
(μg/ml)
Control
LPS
50
100
200
TLR-2

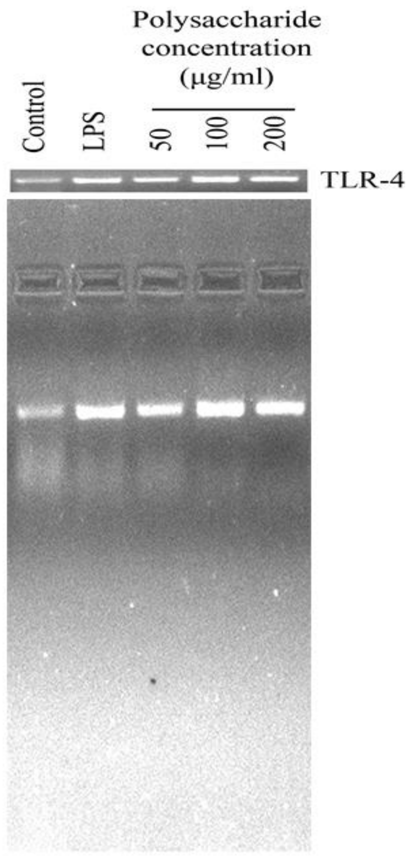

Polysaccharide
concentration
(μg/ml)
Control
LPS
50
100
200
TLR-4

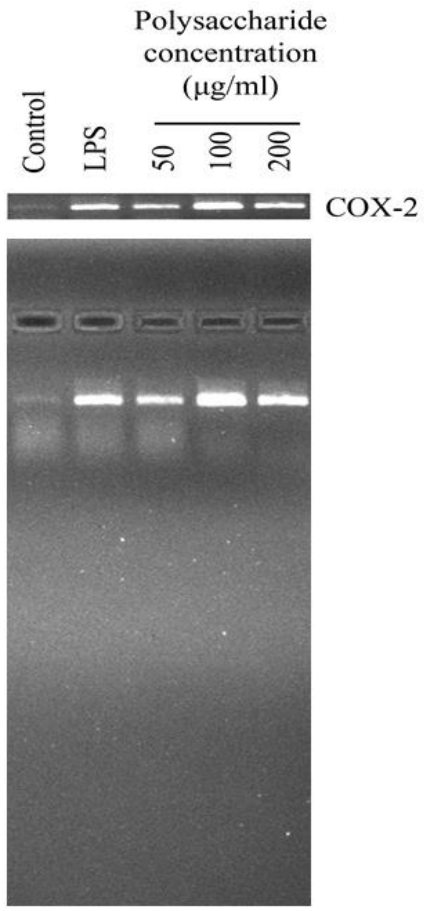

Polysaccharide
concentration
(μg/ml)
Control
LPS
50
100
200
COX-2

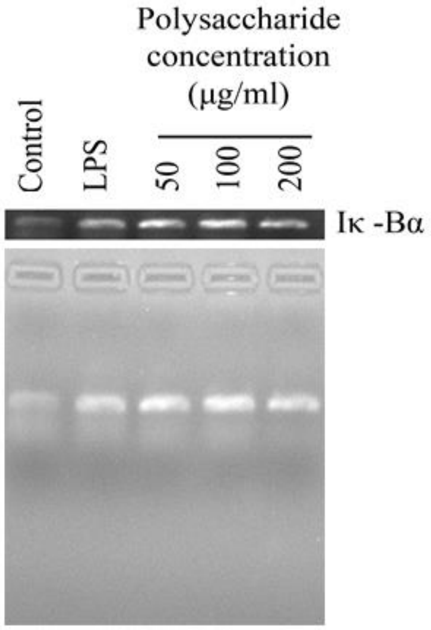
Polysaccharide
concentration
(μg/ml)
Control
LPS
50
100
200
Iκ -Bα

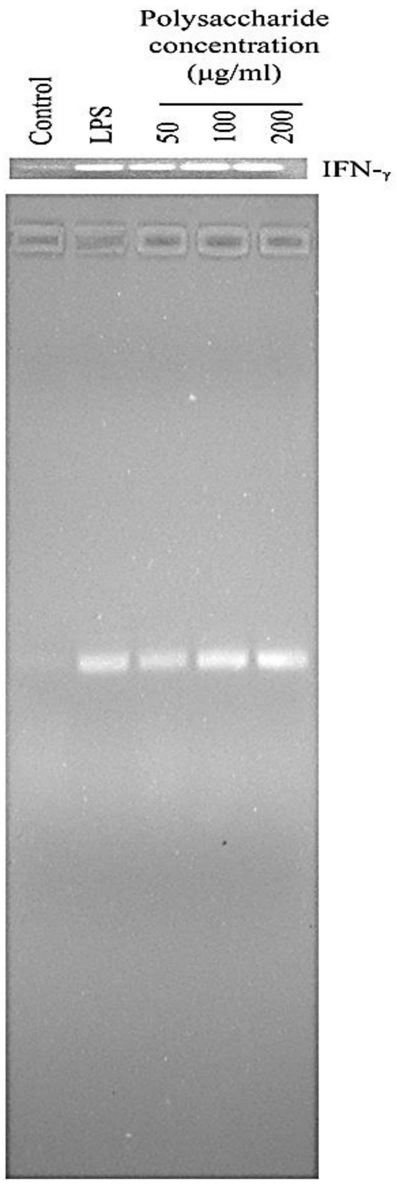
Polysaccharide concentration (μg/ml)
Control
LPS
50
100
200
IFN-γ
